# Optimization of biotechnological systems through geometric programming

**DOI:** 10.1186/1742-4682-4-38

**Published:** 2007-09-26

**Authors:** Alberto Marin-Sanguino, Eberhard O Voit, Carlos Gonzalez-Alcon, Nestor V Torres

**Affiliations:** 1 Grupo de Tecnologia Bioquímica. Departamento de Bioquimica y Biologia Molecular, Facultad de Biologia, Universidad de La Laguna, 38206 La Laguna, Tenerife, Islas Canarias, Spain; 2 The Wallace H. Coulter Department of Biomedical Engineering at Georgia Institute of Technology and Emory University, 313 Ferst Drive, Atlanta, GA, 30332, USA; 3 Grupo de Tecnologia Bioquimica. Departamento de Estadistica Investigacion Operativa y Computacion, Facultad de Fisica y Matematicas, Universidad de La Laguna, 38206 La Laguna, Tenerife, Islas Canarias, Spain

## Abstract

**Background:**

In the past, tasks of model based yield optimization in metabolic engineering were either approached with stoichiometric models or with structured nonlinear models such as S-systems or linear-logarithmic representations. These models stand out among most others, because they allow the optimization task to be converted into a linear program, for which efficient solution methods are widely available. For pathway models not in one of these formats, an Indirect Optimization Method (IOM) was developed where the original model is sequentially represented as an S-system model, optimized in this format with linear programming methods, reinterpreted in the initial model form, and further optimized as necessary.

**Results:**

A new method is proposed for this task. We show here that the model format of a Generalized Mass Action (GMA) system may be optimized very efficiently with techniques of geometric programming. We briefly review the basics of GMA systems and of geometric programming, demonstrate how the latter may be applied to the former, and illustrate the combined method with a didactic problem and two examples based on models of real systems. The first is a relatively small yet representative model of the anaerobic fermentation pathway in *S. cerevisiae*, while the second describes the dynamics of the tryptophan operon in *E. coli*. Both models have previously been used for benchmarking purposes, thus facilitating comparisons with the proposed new method. In these comparisons, the geometric programming method was found to be equal or better than the earlier methods in terms of successful identification of optima and efficiency.

**Conclusion:**

GMA systems are of importance, because they contain stoichiometric, mass action and S-systems as special cases, along with many other models. Furthermore, it was previously shown that algebraic equivalence transformations of variables are sufficient to convert virtually any types of dynamical models into the GMA form. Thus, efficient methods for optimizing GMA systems have multifold appeal.

## Background

Model based optimization of biotechnological processes is a key step towards the establishment of rational strategies for yield improvement, be it through genetic engineering, refined setting of operating conditions or both. As such, it is a key element in the rapidly emerging field of metabolic engineering [1,2]. Optimization tasks involving living organisms are notoriously difficult, because they almost always involve large numbers of variables, representing biological components that dominate cell operation, and must account for multitudinous and complex nonlinear interactions among them [3]. The steady increase in the ready availability of computing power has somewhat alleviated the challenge, but it has also, together with other technological breakthroughs, been raising the level of expectation. Specifically, modelers are more and more expected to account for complex biological details and to include variables of diverse types and origins (metabolites, RNA, proteins...). This trend is to be welcomed, because it promises improved model predictions, yet it easily compensates for the computer technological advances and often overwhelms available hardware and software methods. As a remedy, effort has been expanded to develop computationally efficient algorithms that scale well with the growing number of variables in typical optimization tasks.

The most straightforward attempts toward improved efficiency have been based, in one form or another, on the reduction of the originally nonlinear task to linearity, because linear optimization tasks are rather easily solved, even if they involve thousands of variables. One variant of this approach is the optimization of stoichiometric flux distribution models [4]. The two great advantages of this method are that the models are linear and that minimal information is needed to implement them, namely flux rates, and potentially numerical values characterizing metabolic or physico-chemical constraints. The significant disadvantage is that no regulation can be considered in these models.

An alternative is the use of S-system models within the modeling framework of Biochemical Systems Theory [5-7]. These models are highly nonlinear, thus allowing suitable representations of regulatory features, but have linear steady-state equations, so that optimization under steady-state conditions again becomes a matter of linear programming [8]. The disadvantages here are that much more (kinetic) information is needed to set up numerical models and that S-systems are based on approximations that are not always accepted as valid. Linear-logarithmic models [9] similarly have the advantage of linearity at steady state and the disadvantage of being a local approximation.

An extension of these linear approaches is the Indirect Optimization Method [10]. In this method, any type of kinetic model is locally represented as an S-system. This S-system is optimized with linear methods, and the resulting optimized parameter settings are translated back into the original model. If necessary, this linearized optimization may be executed in sequential steps.

An alternative to using S-system models is the General Mass Action (GMA) representation within BST. GMA systems are very interesting for several reasons. First, they contain both stoichiometric and S-system models as direct special cases, which would allow the optimization of combinations of the two. Second, mass action systems are special cases of GMA models, so that, in some sense, Michaelis-Menten functions and other kinetic rate laws are special cases, if they are expressed in their elemental, non-approximated form. Third, it was shown that virtually any system of differential equations may be represented exactly as a GMA system, upon equivalence transformations of some of the functions in the original system. Thus, GMA systems, as a mathematical representation, are capable of capturing any differentiable nonlinearity that one might encounter in biological systems. We show here that GMA systems, while highly nonlinear, are structured enough to permit the application of efficient optimization methods based on geometric programming.

### Formulation of the optimization task

Pertinent optimization problems in metabolic engineering can be stated as the targeted manipulation of a system in the following way:

max *or *min   *f*_0_(*X*)

subject to:

opearation in steady state

metabolic and physico-chemical constraints

cell viability

In this generic representation, (1) usually targets a flux or a yield. The optimization must occur under several constraints. The first set (2) ensures that the system will operate under steady-state conditions. Other constraints (3) are imposed to retain the system within a physically and chemically feasible state and so that the total protein or metabolite levels do not impede cell growth. Yet other constraints (4) guarantee that no metabolites are depleted below minimal required levels or accumulate to toxic concentrations. These sets of constraints are designed to allow sustained operation of the system.

### Biochemical Systems Theory (BST)

Biological processes are usually modeled as systems of differential equations in which the variation in metabolites **X **is represented as:

dXdt=N⋅v
 MathType@MTEF@5@5@+=feaafiart1ev1aaatCvAUfKttLearuWrP9MDH5MBPbIqV92AaeXatLxBI9gBaebbnrfifHhDYfgasaacH8akY=wiFfYdH8Gipec8Eeeu0xXdbba9frFj0=OqFfea0dXdd9vqai=hGuQ8kuc9pgc9s8qqaq=dirpe0xb9q8qiLsFr0=vr0=vr0dc8meaabaqaciaacaGaaeqabaqabeGadaaakeaadaWcaaqaaiabdsgaKHqabiab=HfaybqaaiabdsgaKjabdsha0baacqGH9aqpcqWGobGtcqGHflY1cqWF2bGDaaa@37F4@

The elements *n*_*i*,*j *_of the stoichiometric matrix *N *are constant. The vector **v **contains reaction rates, which are in general functions of the variables and parameters of the system. This structure is usually associated with metabolic systems, but it is similarly valid for models describing gene expression, bioreactors, and a wide variety of other processes in biotechnology. In typical stoichiometric analyses, the reaction rates are considered constant. Furthermore, the analysis is restricted to steady-state operation, with the consequence that (5) is set equal to 0 and thereby becomes a set of linear algebraic equations, which are amenable to a huge repertoire of analyses.

In analyses accounting for regulation, the reaction rates become functions that depend on system variables and outside influences. Even at steady state, these may be very complex, thereby rendering direct analysis of the system a formidable task [11]. As a remedy, BST suggests to represent these rate functions with power laws:

vi=γi∏j=1n+mXjfi,j
 MathType@MTEF@5@5@+=feaafiart1ev1aaatCvAUfKttLearuWrP9MDH5MBPbIqV92AaeXatLxBI9gBaebbnrfifHhDYfgasaacH8akY=wiFfYdH8Gipec8Eeeu0xXdbba9frFj0=OqFfea0dXdd9vqai=hGuQ8kuc9pgc9s8qqaq=dirpe0xb9q8qiLsFr0=vr0=vr0dc8meaabaqaciaacaGaaeqabaqabeGadaaakeaacqWG2bGDdaWgaaWcbaGaemyAaKgabeaakiabg2da9GGaciab=n7aNnaaBaaaleaacqWGPbqAaeqaaOWaaebCaeaacqWGybawdaqhaaWcbaGaemOAaOgabaGaemOzay2aaSbaaWqaaiabdMgaPjabcYcaSiabdQgaQbqabaaaaaWcbaGaemOAaOMaeyypa0JaeGymaedabaGaemOBa4Maey4kaSIaemyBa0ganiabg+Givdaaaa@4502@

In analogy with chemical kinetics, *γ*_*i *_is called the rate constant and *f*_*i*,*j *_are kinetic orders, which may be any real numbers. Positive kinetic orders indicate augmentation, whereas negative values are indicative of inhibition. Kinetic orders of 0 result in automatic removal of the corresponding variable from the term. In the notation of BST, the first *n *variables are often considered the *dependent variables*, which change dynamically under the action of the system, while the remaining variables *X*_*i *_for *i *= *n *+ 1 ... *m *+ *n *are considered *independent variables *and typically remain constant throughout any given simulation study. Thus, metabolites, enzymes, membrane potentials or other system components can easily be made dependent or independent by the modeler without requiring alterations in the structure of the equations. BST is very compact and explicitly distinguishes variables from parameters.

Because we will later introduce concepts of geometric programming, it is noted that the power-law term in Eq. 6 is also called a *monomial*. If this monomial is an approximation of reaction rate *V*, its parameters can be directly related to *V*, by virtue of the fact that the monomial is in fact a Taylor linearization in logarithmic space [12]. Thus, choosing an operating point with index 0, one obtains:

ln⁡vi=ln⁡V0+|∂ln⁡V∂ln⁡X1|0(ln⁡X1−|ln⁡X1|0)+⋯+|∂ln⁡V∂ln⁡Xm+n|0(ln⁡Xm+n−|ln⁡Xm+n|0)
 MathType@MTEF@5@5@+=feaafiart1ev1aaatCvAUfKttLearuWrP9MDH5MBPbIqV92AaeXatLxBI9gBaebbnrfifHhDYfgasaacH8akY=wiFfYdH8Gipec8Eeeu0xXdbba9frFj0=OqFfea0dXdd9vqai=hGuQ8kuc9pgc9s8qqaq=dirpe0xb9q8qiLsFr0=vr0=vr0dc8meaabaqaciaacaGaaeqabaqabeGadaaakeaacyGGSbaBcqGGUbGBcqWG2bGDdaWgaaWcbaGaemyAaKgabeaakiabg2da9iGbcYgaSjabc6gaUjabdAfawnaaBaaaleaacqaIWaamaeqaaOGaey4kaSYaaqWaaeaadaWcaaqaaiabgkGi2kGbcYgaSjabc6gaUjabdAfawbqaaiabgkGi2kGbcYgaSjabc6gaUjabdIfaynaaBaaaleaacqaIXaqmaeqaaaaaaOGaay5bSlaawIa7amaaBaaaleaacqaIWaamaeqaaOWaaeWaaeaacyGGSbaBcqGGUbGBcqWGybawdaWgaaWcbaGaeGymaedabeaakiabgkHiTmaaemaabaGagiiBaWMaeiOBa4MaemiwaG1aaSbaaSqaaiabigdaXaqabaaakiaawEa7caGLiWoadaWgaaWcbaGaeGimaadabeaaaOGaayjkaiaawMcaaiabgUcaRiabl+UimjabgUcaRmaaemaabaWaaSaaaeaacqGHciITcyGGSbaBcqGGUbGBcqWGwbGvaeaacqGHciITcyGGSbaBcqGGUbGBcqWGybawdaWgaaWcbaGaemyBa0Maey4kaSIaemOBa4gabeaaaaaakiaawEa7caGLiWoadaWgaaWcbaGaeGimaadabeaakmaabmaabaGagiiBaWMaeiOBa4MaemiwaG1aaSbaaSqaaiabd2gaTjabgUcaRiabd6gaUbqabaGccqGHsisldaabdaqaaiGbcYgaSjabc6gaUjabdIfaynaaBaaaleaacqWGTbqBcqGHRaWkcqWGUbGBaeqaaaGccaGLhWUaayjcSdWaaSbaaSqaaiabicdaWaqabaaakiaawIcacaGLPaaaaaa@87EF@

Thus, it follows directly from 7 that the parameters of a power-law (monomial) term can be computed as

γi=|vi|0|∏j=1n+mXjfi,j|0
MathType@MTEF@5@5@+=feaafiart1ev1aaatCvAUfKttLearuWrP9MDH5MBPbIqV92AaeXatLxBI9gBaebbnrfifHhDYfgasaacH8akY=wiFfYdH8Gipec8Eeeu0xXdbba9frFj0=OqFfea0dXdd9vqai=hGuQ8kuc9pgc9s8qqaq=dirpe0xb9q8qiLsFr0=vr0=vr0dc8meaabaqaciaacaGaaeqabaqabeGadaaakeaaiiGacqWFZoWzdaWgaaWcbaGaemyAaKgabeaakiabg2da9maalaaabaWaaqWaaeaacqWG2bGDdaWgaaWcbaGaemyAaKgabeaaaOGaay5bSlaawIa7amaaBaaaleaacqaIWaamaeqaaaGcbaWaaqWaaeaadaqeWaqaaiabdIfaynaaDaaaleaacqWGQbGAaeaacqWGMbGzdaWgaaadbaGaemyAaKMaeiilaWIaemOAaOgabeaaaaaaleaacqWGQbGAcqGH9aqpcqaIXaqmaeaacqWGUbGBcqGHRaWkcqWGTbqBa0Gaey4dIunaaOGaay5bSlaawIa7amaaBaaaleaacqaIWaamaeqaaaaaaaa@4D5E@

fi,j=|∂ln⁡vi∂ln⁡Xj|0=|∂vi∂XjXjvi|0
 MathType@MTEF@5@5@+=feaafiart1ev1aaatCvAUfKttLearuWrP9MDH5MBPbIqV92AaeXatLxBI9gBaebbnrfifHhDYfgasaacH8akY=wiFfYdH8Gipec8Eeeu0xXdbba9frFj0=OqFfea0dXdd9vqai=hGuQ8kuc9pgc9s8qqaq=dirpe0xb9q8qiLsFr0=vr0=vr0dc8meaabaqaciaacaGaaeqabaqabeGadaaakeaacqWGMbGzdaWgaaWcbaGaemyAaKMaeiilaWIaemOAaOgabeaakiabg2da9maaemaabaWaaSaaaeaacqGHciITcyGGSbaBcqGGUbGBcqWG2bGDdaWgaaWcbaGaemyAaKgabeaaaOqaaiabgkGi2kGbcYgaSjabc6gaUjabdIfaynaaBaaaleaacqWGQbGAaeqaaaaaaOGaay5bSlaawIa7amaaBaaaleaacqaIWaamaeqaaOGaeyypa0ZaaqWaaeaadaWcaaqaaiabgkGi2kabdAha2naaBaaaleaacqWGPbqAaeqaaaGcbaGaeyOaIyRaemiwaG1aaSbaaSqaaiabdQgaQbqabaaaaOWaaSaaaeaacqWGybawdaWgaaWcbaGaemOAaOgabeaaaOqaaiabdAha2naaBaaaleaacqWGPbqAaeqaaaaaaOGaay5bSlaawIa7amaaBaaaleaacqaIWaamaeqaaaaa@5927@

System equations in BST may be designed in slightly different ways. For the GMA form, each reaction is represented by its own monomial, and the result is therefore

dXidt=∑j=1pni,jγj∏k=1n+mXkfj,ki=1...n
 MathType@MTEF@5@5@+=feaafiart1ev1aaatCvAUfKttLearuWrP9MDH5MBPbIqV92AaeXatLxBI9gBaebbnrfifHhDYfgasaacH8akY=wiFfYdH8Gipec8Eeeu0xXdbba9frFj0=OqFfea0dXdd9vqai=hGuQ8kuc9pgc9s8qqaq=dirpe0xb9q8qiLsFr0=vr0=vr0dc8meaabaqaciaacaGaaeqabaqabeGadaaakeaafaqabeqacaaabaWaaSaaaeaacqWGKbazcqWGybawdaWgaaWcbaGaemyAaKgabeaaaOqaaiabdsgaKjabdsha0baacqGH9aqpdaaeWbqaaiabd6gaUnaaBaaaleaacqWGPbqAcqGGSaalcqWGQbGAaeqaaGGacOGae83SdC2aaSbaaSqaaiabdQgaQbqabaaabaGaemOAaOMaeyypa0JaeGymaedabaGaemiCaahaniabggHiLdGcdaqeWbqaaiabdIfaynaaDaaaleaacqWGRbWAaeaacqWGMbGzdaWgaaadbaGaemOAaOMaeiilaWIaem4AaSgabeaaaaaaleaacqWGRbWAcqGH9aqpcqaIXaqmaeaacqWGUbGBcqGHRaWkcqWGTbqBa0Gaey4dIunaaOqaaiabdMgaPjabg2da9iabigdaXiabc6caUiabc6caUiabc6caUiabd6gaUbaaaaa@5C92@

Note that this is actually a spelled-out version of Eq. 5, where the reaction rates are monomials as in Eq. 6. As an alternative to the GMA format, one may, for each dependent variable, collect all incoming reactions in one term Vi+
 MathType@MTEF@5@5@+=feaafiart1ev1aaatCvAUfKttLearuWrP9MDH5MBPbIqV92AaeXatLxBI9gBaebbnrfifHhDYfgasaacH8akY=wiFfYdH8Gipec8Eeeu0xXdbba9frFj0=OqFfea0dXdd9vqai=hGuQ8kuc9pgc9s8qqaq=dirpe0xb9q8qiLsFr0=vr0=vr0dc8meaabaqaciaacaGaaeqabaqabeGadaaakeaacqWGwbGvdaqhaaWcbaGaemyAaKgabaGaey4kaScaaaaa@304B@ and do the same with all outgoing fluxes, which are collectively called Vi−
 MathType@MTEF@5@5@+=feaafiart1ev1aaatCvAUfKttLearuWrP9MDH5MBPbIqV92AaeXatLxBI9gBaebbnrfifHhDYfgasaacH8akY=wiFfYdH8Gipec8Eeeu0xXdbba9frFj0=OqFfea0dXdd9vqai=hGuQ8kuc9pgc9s8qqaq=dirpe0xb9q8qiLsFr0=vr0=vr0dc8meaabaqaciaacaGaaeqabaqabeGadaaakeaacqWGwbGvdaqhaaWcbaGaemyAaKgabaGaeyOeI0caaaaa@3056@. These *aggregated *terms are now represented as monomials, and the result is

dXidt=Vi+−Vi−=αi∏j=1n+mXjgi,j−βi∏j=1n+mXjhi,j
 MathType@MTEF@5@5@+=feaafiart1ev1aaatCvAUfKttLearuWrP9MDH5MBPbIqV92AaeXatLxBI9gBaebbnrfifHhDYfgasaacH8akY=wiFfYdH8Gipec8Eeeu0xXdbba9frFj0=OqFfea0dXdd9vqai=hGuQ8kuc9pgc9s8qqaq=dirpe0xb9q8qiLsFr0=vr0=vr0dc8meaabaqaciaacaGaaeqabaqabeGadaaakeaadaWcaaqaaiabdsgaKjabdIfaynaaBaaaleaacqWGPbqAaeqaaaGcbaGaemizaqMaemiDaqhaaiabg2da9iabdAfawnaaDaaaleaacqWGPbqAaeaacqGHRaWkaaGccqGHsislcqWGwbGvdaqhaaWcbaGaemyAaKgabaGaeyOeI0caaOGaeyypa0dcciGae8xSde2aaSbaaSqaaiabdMgaPbqabaGcdaqeWbqaaiabdIfaynaaDaaaleaacqWGQbGAaeaacqWGNbWzdaWgaaadbaGaemyAaKMaeiilaWIaemOAaOgabeaaaaaaleaacqWGQbGAcqGH9aqpcqaIXaqmaeaacqWGUbGBcqGHRaWkcqWGTbqBa0Gaey4dIunakiabgkHiTiab=j7aInaaBaaaleaacqWGPbqAaeqaaOWaaebCaeaacqWGybawdaqhaaWcbaGaemOAaOgabaGaemiAaG2aaSbaaWqaaiabdMgaPjabcYcaSiabdQgaQbqabaaaaaWcbaGaemOAaOMaeyypa0JaeGymaedabaGaemOBa4Maey4kaSIaemyBa0ganiabg+Givdaaaa@6766@

Thus, there are at most one positive and one negative term in each S-system equation.

The conversion of a GMA into an S-system will become important later. It is achieved by collecting the aggregated fluxes into vectors

V+=N+vV−=N−v
 MathType@MTEF@5@5@+=feaafiart1ev1aaatCvAUfKttLearuWrP9MDH5MBPbIqV92AaeXatLxBI9gBaebbnrfifHhDYfgasaacH8akY=wiFfYdH8Gipec8Eeeu0xXdbba9frFj0=OqFfea0dXdd9vqai=hGuQ8kuc9pgc9s8qqaq=dirpe0xb9q8qiLsFr0=vr0=vr0dc8meaabaqaciaacaGaaeqabaqabeGadaaakeaafaqabeGabaaabaacbeGae8Nvay1aaWbaaSqabeaacqGHRaWkaaGccqGH9aqpcqWGobGtdaahaaWcbeqaaiabgUcaRaaakiab=zha2bqaaiab=zfawnaaCaaaleqabaGaeyOeI0caaOGaeyypa0JaemOta40aaWbaaSqabeaacqGHsislaaGccqWF2bGDaaaaaa@3AD7@

where *N*^+ ^and *N*^- ^are matrices containing respectively the positive and negative coefficients of *N *such that *N *= *N*^+ ^- *N*^-^. With these definitions, we can derive the matrices of kinetic orders of S-systems from those of the corresponding GMA representation. Namely,

G=(V+)−1N+VFH=(V−)−1N−VF
 MathType@MTEF@5@5@+=feaafiart1ev1aaatCvAUfKttLearuWrP9MDH5MBPbIqV92AaeXatLxBI9gBaebbnrfifHhDYfgasaacH8akY=wiFfYdH8Gipec8Eeeu0xXdbba9frFj0=OqFfea0dXdd9vqai=hGuQ8kuc9pgc9s8qqaq=dirpe0xb9q8qiLsFr0=vr0=vr0dc8meaabaqaciaacaGaaeqabaqabeGadaaakeaafaqadeGabaaabaGaem4raCKaeyypa0JaeiikaGIaemOvay1aaWbaaSqabeaacqGHRaWkaaGccqGGPaqkdaahaaWcbeqaaiabgkHiTiabigdaXaaakiabd6eaonaaCaaaleqabaGaey4kaScaaGqabOGae8NvayLaemOrayeabaGaemisaGKaeyypa0JaeiikaGIaemOvay1aaWbaaSqabeaacqGHsislaaGccqGGPaqkdaahaaWcbeqaaiabgkHiTiabigdaXaaakiabd6eaonaaCaaaleqabaGaeyOeI0caaOGae8NvayLaemOrayeaaaaa@4647@

where **V**, **V**^+ ^and **V**^- ^are square matrices of zeros having the corresponding vectors as their main diagonals. G and H contain the kinetic orders of the S-system while F contains those of the GMA [13]. GMA systems may be constructed in three manners [11]. First, given a pathway diagram, each reaction rate is represented by a monomial, and equations are assembled from all reaction rates involved. Second, it is possible (though not often actually done) to dissect enzyme catalyzed reactions into their underlying mass action kinetics, without evoking the typical quasi-steady-state assumption. The result is directly the special case of a GMA system where most kinetic orders are zero, one, or in some cases 2. Third, it has been shown that virtually any nonlinearity can be represented equivalently as a GMA system [14]. As an example for this *recasting *technique, consider a simple equation where production and degradation are formulated as traditional Michaelis-Menten rate laws:



where *X*_0 _is a dependent or independent variable describing the substrate for the generation of *X*_1_. To effect the transformation into a GMA equation, define auxiliary variables as *X*_2 _= *K*_*M*,2 _+ *X*_1 _and *X*_3 _= *K*_*M*,1 _+ *X*_0_. The equation then becomes

dX1dt=Vmax,1X0X3−1−Vmax,2X1X2−1
 MathType@MTEF@5@5@+=feaafiart1ev1aaatCvAUfKttLearuWrP9MDH5MBPbIqV92AaeXatLxBI9gBaebbnrfifHhDYfgasaacH8akY=wiFfYdH8Gipec8Eeeu0xXdbba9frFj0=OqFfea0dXdd9vqai=hGuQ8kuc9pgc9s8qqaq=dirpe0xb9q8qiLsFr0=vr0=vr0dc8meaabaqaciaacaGaaeqabaqabeGadaaakeaadaWcaaqaaiabdsgaKjabdIfaynaaBaaaleaacqaIXaqmaeqaaaGcbaGaemizaqMaemiDaqhaaiabg2da9iabdAfawnaaBaaaleaacqWGTbqBcqWGHbqycqWG4baEcqGGSaalcqaIXaqmaeqaaOGaemiwaG1aaSbaaSqaaiabicdaWaqabaGccqWGybawdaqhaaWcbaGaeG4mamdabaGaeyOeI0IaeGymaedaaOGaeyOeI0IaemOvay1aaSbaaSqaaiabd2gaTjabdggaHjabdIha4jabcYcaSiabikdaYaqabaGccqWGybawdaWgaaWcbaGaeGymaedabeaakiabdIfaynaaDaaaleaacqaIYaGmaeaacqGHsislcqaIXaqmaaaaaa@5119@

For simplicity of discussion, suppose that *X*_0 _is a constant, independent variable. Thus, *X*_3 _is also constant and does not need its own equation. By contrast, *X*_2 _is a new dependent variable and from its definition we can calculate its initial value and see that its derivative must be equal to that of *X*_1. _Therefore the equations:

dX1dt=Vmax,1X0X3−1−Vmax,2X1X2−1dX2dt=Vmax,1X0X3−1−Vmax,2X1X2−1X1(t0)=X10X2(t0)=KM,2+X10
 MathType@MTEF@5@5@+=feaafiart1ev1aaatCvAUfKttLearuWrP9MDH5MBPbIqV92AaeXatLxBI9gBaebbnrfifHhDYfgasaacH8akY=wiFfYdH8Gipec8Eeeu0xXdbba9frFj0=OqFfea0dXdd9vqai=hGuQ8kuc9pgc9s8qqaq=dirpe0xb9q8qiLsFr0=vr0=vr0dc8meaabaqaciaacaGaaeqabaqabeGadaaakeaafaqadeabbaaaaeaadaWcaaqaaiabdsgaKjabdIfaynaaBaaaleaacqaIXaqmaeqaaaGcbaGaemizaqMaemiDaqhaaiabg2da9iabdAfawnaaBaaaleaacqWGTbqBcqWGHbqycqWG4baEcqGGSaalcqaIXaqmaeqaaOGaemiwaG1aaSbaaSqaaiabicdaWaqabaGccqWGybawdaqhaaWcbaGaeG4mamdabaGaeyOeI0IaeGymaedaaOGaeyOeI0IaemOvay1aaSbaaSqaaiabd2gaTjabdggaHjabdIha4jabcYcaSiabikdaYaqabaGccqWGybawdaWgaaWcbaGaeGymaedabeaakiabdIfaynaaDaaaleaacqaIYaGmaeaacqGHsislcqaIXaqmaaaakeaadaWcaaqaaiabdsgaKjabdIfaynaaBaaaleaacqaIYaGmaeqaaaGcbaGaemizaqMaemiDaqhaaiabg2da9iabdAfawnaaBaaaleaacqWGTbqBcqWGHbqycqWG4baEcqGGSaalcqaIXaqmaeqaaOGaemiwaG1aaSbaaSqaaiabicdaWaqabaGccqWGybawdaqhaaWcbaGaeG4mamdabaGaeyOeI0IaeGymaedaaOGaeyOeI0IaemOvay1aaSbaaSqaaiabd2gaTjabdggaHjabdIha4jabcYcaSiabikdaYaqabaGccqWGybawdaWgaaWcbaGaeGymaedabeaakiabdIfaynaaDaaaleaacqaIYaGmaeaacqGHsislcqaIXaqmaaaakeaacqWGybawdaWgaaWcbaGaeGymaedabeaakiabcIcaOiabdsha0naaBaaaleaacqaIWaamaeqaaOGaeiykaKIaeyypa0JaemiwaG1aa0baaSqaaiabigdaXaqaaiabicdaWaaaaOqaaiabdIfaynaaBaaaleaacqaIYaGmaeqaaOGaeiikaGIaemiDaq3aaSbaaSqaaiabicdaWaqabaGccqGGPaqkcqGH9aqpcqWGlbWsdaWgaaWcbaGaemyta0KaeiilaWIaeGOmaidabeaakiabgUcaRiabdIfaynaaDaaaleaacqaIXaqmaeaacqaIWaamaaaaaaaa@90C7@

form a system that is an exact equivalent of the original system but in GMA format.

Recasting can be useful with equations that are difficult to handle otherwise or for purposes of streamlining a model structure and its analysis. One must note though that often the number of variables increases significantly. In the case shown, the number of equations rises from one to two if *X*_0 _is independent or to three if it is a dependent variable.

### Current optimization methods based on BST

The overall task is to reset some of the independent variables so that some objective is optimized. The independent variables in question are typically enzyme activities, which are experimentally manipulated through genetic means, such as the application of customized promoters or plasmids. The objective is usually the maximization of a metabolite concentration or a flux. Three approaches have been proposed in the literature.

#### Pure S-systems

Among a number of convenient properties, the steady states of an S-system can be computed analytically by solving a system of algebraic linear equation [6]. Equating Eq. 11 to zero and rearranging one obtains:

αi∏j=1nXjgi,jβi∏j=1nXjhi,j=1
MathType@MTEF@5@5@+=feaafiart1ev1aaatCvAUfKttLearuWrP9MDH5MBPbIqV92AaeXatLxBI9gBaebbnrfifHhDYfgasaacH8akY=wiFfYdH8Gipec8Eeeu0xXdbba9frFj0=OqFfea0dXdd9vqai=hGuQ8kuc9pgc9s8qqaq=dirpe0xb9q8qiLsFr0=vr0=vr0dc8meaabaqaciaacaGaaeqabaqabeGadaaakeaadaWcaaqaaGGaciab=f7aHnaaBaaaleaacqWGPbqAaeqaaOWaaebmaeaacqWGybawdaqhaaWcbaGaemOAaOgabaGaem4zaC2aaSbaaWqaaiabdMgaPjabcYcaSiabdQgaQbqabaaaaaWcbaGaemOAaOMaeyypa0JaeGymaedabaGaemOBa4ganiabg+GivdaakeaacqWFYoGydaWgaaWcbaGaemyAaKgabeaakmaaradabaGaemiwaG1aa0baaSqaaiabdQgaQbqaaiabdIgaOnaaBaaameaacqWGPbqAcqGGSaalcqWGQbGAaeqaaaaaaSqaaiabdQgaQjabg2da9iabigdaXaqaaiabd6gaUbqdcqGHpis1aaaakiabg2da9iabigdaXaaa@523C@

which is a monomial of the form

αiβi∏j=1nXjgi,j−hi,j=1.
 MathType@MTEF@5@5@+=feaafiart1ev1aaatCvAUfKttLearuWrP9MDH5MBPbIqV92AaeXatLxBI9gBaebbnrfifHhDYfgasaacH8akY=wiFfYdH8Gipec8Eeeu0xXdbba9frFj0=OqFfea0dXdd9vqai=hGuQ8kuc9pgc9s8qqaq=dirpe0xb9q8qiLsFr0=vr0=vr0dc8meaabaqaciaacaGaaeqabaqabeGadaaakeaadaWcaaqaaGGaciab=f7aHnaaBaaaleaacqWGPbqAaeqaaaGcbaGae8NSdi2aaSbaaSqaaiabdMgaPbqabaaaaOWaaebCaeaacqWGybawdaqhaaWcbaGaemOAaOgabaGaem4zaC2aaSbaaWqaaiabdMgaPjabcYcaSiabdQgaQbqabaWccqGHsislcqWGObaAdaWgaaadbaGaemyAaKMaeiilaWIaemOAaOgabeaaaaaaleaacqWGQbGAcqGH9aqpcqaIXaqmaeaacqWGUbGBa0Gaey4dIunakiabg2da9iabigdaXiabc6caUaaa@4AE2@

Monomial equations become linear by taking logarithms on both sides thus reducing the steady-state computation to a linear task:

A·**y **= **b**

where

*A*_*i*,*j *_= *g*_*i*,*j *_- *h*_*i*,*j*_

*y*_*i *_= In *X*_*i*_

bi=ln⁡βiαi
 MathType@MTEF@5@5@+=feaafiart1ev1aaatCvAUfKttLearuWrP9MDH5MBPbIqV92AaeXatLxBI9gBaebbnrfifHhDYfgasaacH8akY=wiFfYdH8Gipec8Eeeu0xXdbba9frFj0=OqFfea0dXdd9vqai=hGuQ8kuc9pgc9s8qqaq=dirpe0xb9q8qiLsFr0=vr0=vr0dc8meaabaqaciaacaGaaeqabaqabeGadaaakeaacqWGIbGydaWgaaWcbaGaemyAaKgabeaakiabg2da9iGbcYgaSjabc6gaUnaalaaabaacciGae8NSdi2aaSbaaSqaaiabdMgaPbqabaaakeaacqWFXoqydaWgaaWcbaGaemyAaKgabeaaaaaaaa@39C0@

Monomial objective functions become linear by taking logarithms and so holds for many constraints on metabolites or fluxes. Therefore, constrained optimization of pathways modeled as S-systems becomes a straightforward linear program [8].

Any other relevant constraint or objective function that is not a power law can also be approximated using the abovementioned methods. Then logarithms can be taken and Eqns 1–4 can be rewritten as:

max *or *min   *F*(**y**)

Subject to:

*A*·**y **= **b**

*B*·**y **= **d**

*C*·**y **≤ **e**

**y**^**L **^≤ **y **≤ **y**^**U**^

Where *F *is the logarithm of the flux or variable to be optimized, and superscripts *L *and *U *refer to lower and upper bounds. Eq. 20 assures operation at steady state. Matrix *B *and vector **d **account for additional equality constraints and *C *and **e **are analogous constraints for additional inequalities, which could, for instance, limit the magnitude of a metabolite concentration or flux, and improve the chances of viability. Optimization problems of this type are called *linear programs *(LPs) and can be solved very efficiently for large numbers of variables and constraints [15].

The advantage of the pure S-system approach is its great speed combined with the fact that S-system models have proven to be excellent representations of many pathways. The disadvantage is that the optimization process, by design, moves the system away from the chosen operating point, so that questions arise as to how accurate the S-system representation is at the steady state suggested by the optimization.

#### Indirect Optimization Method

If the pathway is not modeled as an S-system, the reduction of the optimization task to linearity is jeopardized. A compromise solution that has turned out to be quite effective is the Indirect Optimization Method (IOM) [10]. The first step of IOM is approximation of the alleged model with an S-system. This S-system is optimized as shown above. The solution is then translated back into the original system in order to confirm that it constitutes a stable steady state and is really an improvement from the basal state of the original model. The S-system solution typically differs somewhat from a direct optimization result with the original model, but since it is obtained so fast, it is possible to execute IOM in several steps with relatively tight bounds, every time choosing a new operating point and not deviating too much from this point in the next iteration [16]. The speed of the process is slower than in the pure S-system case, but still reasonable. Variations on IOM are to search for subsets of independent variables to be manipulated for optimal yield at lower cost and for multi-objective optimization tasks [17,18].

#### Global GMA optimization

A global optimization method for GMA systems [19] has been recently proposed based on branch-and-reduce methods combined with convexification. These methods are interesting because of the variety of roles that GMA models can play (see above). The disadvantage of the global method is that it quickly leads to very large systems that are non-convex, even though they allow relatively efficient solutions.

### Geometric programming

Geometric programming (GP) [20] addresses a class of problems that include linear programming (LP) and other tasks within the broader category of convex optimization problems. Convex problems are among the few nonlinear tasks where, thanks to powerful interior point methods, the efficient determination of global optima is feasible even for large scale systems. For example, a geometric program of 1,000 variables and 10,000 constraints can be solved in less than a minute on a desktop computer [21]; the solution is even faster for sparse problems as they are found in metabolic engineering. Furthermore, easy to use solvers are starting to become available [22,23].

GP addresses optimization programs where the objective function and the constraints are sums of monomials, i.e., power-law terms as shown in Eq. 6. Because of their importance in GP, sums of monomials, all with positive sign, are called *posynomials*. If some of the monomials enter the sum with negative signs, the collection is called a *signomial*. The peculiarities of convexity and GP methods render the difference between posynomials and signomials crucial.

A GP problem has the generic form:

min   *P*_0_(**x**)

Subject to:

*P*_*i*_(**x**) ≤ 1 *i *= 1...*n*

*M*_*i*_(**x**) = 1 *i *= 1...*p*

where *P*_*i*_(**x**) and *M*_*i*_(**x**) must fulfill strict conditions. Every function *M*_*i*_(**x**) must be a monomial, while the objective function *P*_0_(**x**) and the functions *P*_*i*_(**x**) involved in inequalities must be posynomials. Signomials are not permitted, and optimization problems involving them require additional effort.

The equivalence between monomials and power laws immediately suggests the potential use of GP for optimization problems formulated within BST. In the next sections, several methods will be proposed to develop such potential.

## Results and discussion

It is easy to see that steady-state equations of S-systems are readily arranged as monomials as shown in Eq 18 and that optimization tasks for S-systems directly adhere to the format of a GP, except that GP mandates minimization. However, this is easily remedied for maximization tasks by minimizing the inverse of the objective, which again is a monomial. By contrast, steady-state GMA equations as shown in Eq. 10 do not automatically fall within the GP structure, because GMA systems usually include negative terms, thus making them signomials. Furthermore, inversion of an objective that contains more than one monomial is not equivalent to a monomial.

When the objective or some restriction falls outside the GMA formalism, it can be recast into proper form as has been discussed above and will be shown in one of the case studies.

### Two strategies

The proposed solutions for adapting GP solvers to treat GMA systems rely on *condensation *[24], but they do it in different ways. Condensation is a standard procedure in GP which is exactly equivalent to aggregation in BST. Namely, the sum of monomials is approximated by a single monomial. In the terminology of GP, the condensation C^()
 MathType@MTEF@5@5@+=feaafiart1ev1aaatCvAUfKttLearuWrP9MDH5MBPbIqV92AaeXatLxBI9gBaebbnrfifHhDYfgasaacH8akY=wiFfYdH8Gipec8Eeeu0xXdbba9frFj0=OqFfea0dXdd9vqai=hGuQ8kuc9pgc9s8qqaq=dirpe0xb9q8qiLsFr0=vr0=vr0dc8meaabaqaciaacaGaaeqabaqabeGadaaakeaacuWGdbWqgaqcaiabcIcaOiabcMcaPaaa@2F7D@ is generically denoted as

C^(P(x)=C^(M1(x)+⋯+Mn(x))=M0(x)
 MathType@MTEF@5@5@+=feaafiart1ev1aaatCvAUfKttLearuWrP9MDH5MBPbIqV92AaeXatLxBI9gBaebbnrfifHhDYfgasaacH8akY=wiFfYdH8Gipec8Eeeu0xXdbba9frFj0=OqFfea0dXdd9vqai=hGuQ8kuc9pgc9s8qqaq=dirpe0xb9q8qiLsFr0=vr0=vr0dc8meaabaqaciaacaGaaeqabaqabeGadaaakeaacuWGdbWqgaqcaiabcIcaOiabdcfaqjabcIcaOGqabiab=Hha4jabcMcaPiabg2da9iqbdoeadzaajaGaeiikaGIaemyta00aaSbaaSqaaiabigdaXaqabaGccqGGOaakcqWF4baEcqGGPaqkcqGHRaWkcqWIVlctcqGHRaWkcqWGnbqtdaWgaaWcbaGaemOBa4gabeaakiabcIcaOiab=Hha4jabcMcaPiabcMcaPiabg2da9iabd2eannaaBaaaleaacqaIWaamaeqaaOGaeiikaGIae8hEaGNaeiykaKcaaa@4C4F@

and, in the terminology of Eqs. 10 and 11, defined as:

C^(∑j=1kni,jγj∏k=1n+mXkfj,k)=αi∏j=1nXjgi,j
 MathType@MTEF@5@5@+=feaafiart1ev1aaatCvAUfKttLearuWrP9MDH5MBPbIqV92AaeXatLxBI9gBaebbnrfifHhDYfgasaacH8akY=wiFfYdH8Gipec8Eeeu0xXdbba9frFj0=OqFfea0dXdd9vqai=hGuQ8kuc9pgc9s8qqaq=dirpe0xb9q8qiLsFr0=vr0=vr0dc8meaabaqaciaacaGaaeqabaqabeGadaaakeaacuWGdbWqgaqcamaabmaabaWaaabCaeaacqWGUbGBdaWgaaWcbaGaemyAaKMaeiilaWIaemOAaOgabeaaiiGakiab=n7aNnaaBaaaleaacqWGQbGAaeqaaaqaaiabdQgaQjabg2da9iabigdaXaqaaiabdUgaRbqdcqGHris5aOWaaebCaeaacqWGybawdaqhaaWcbaGaem4AaSgabaGaemOzay2aaSbaaWqaaiabdQgaQjabcYcaSiabdUgaRbqabaaaaaWcbaGaem4AaSMaeyypa0JaeGymaedabaGaemOBa4Maey4kaSIaemyBa0ganiabg+GivdaakiaawIcacaGLPaaacqGH9aqpcqWFXoqydaWgaaWcbaGaemyAaKgabeaakmaarahabaGaemiwaG1aa0baaSqaaiabdQgaQbqaaiabdEgaNnaaBaaameaacqWGPbqAcqGGSaalcqWGQbGAaeqaaaaaaSqaaiabdQgaQjabg2da9iabigdaXaqaaiabd6gaUbqdcqGHpis1aaaa@62C7@

where *α*_*i *_and *g*_*i*,*j *_are chosen such that equality holds at a chosen operating point; thus, the result is equivalent to the Taylor linearization that is fundamental in BST as was shown in eqn. 7 [5,7,12]. As in the Taylor series, the condensed form is equal to the original equation at the operating point. For any other point, as it can be shown that the left and right hand side of eqn. 29 are equivalent to those of the Arithmetic-Geometric inequality:

∑i=1nai≥∏i=1n(aiwi)wi
 MathType@MTEF@5@5@+=feaafiart1ev1aaatCvAUfKttLearuWrP9MDH5MBPbIqV92AaeXatLxBI9gBaebbnrfifHhDYfgasaacH8akY=wiFfYdH8Gipec8Eeeu0xXdbba9frFj0=OqFfea0dXdd9vqai=hGuQ8kuc9pgc9s8qqaq=dirpe0xb9q8qiLsFr0=vr0=vr0dc8meaabaqaciaacaGaaeqabaqabeGadaaakeaadaaeWbqaaiabdggaHnaaBaaaleaacqWGPbqAaeqaaOGaeyyzIm7aaebCaeaadaqadaqaamaalaaabaGaemyyae2aaSbaaSqaaiabdMgaPbqabaaakeaacqWG3bWDdaWgaaWcbaGaemyAaKgabeaaaaaakiaawIcacaGLPaaadaahaaWcbeqaaiabdEha3naaBaaameaacqWGPbqAaeqaaaaaaSqaaiabdMgaPjabg2da9iabigdaXaqaaiabd6gaUbqdcqGHpis1aaWcbaGaemyAaKMaeyypa0JaeGymaedabaGaemOBa4ganiabggHiLdaaaa@49D6@

and therefore, the condensed form is an understimation of the original.

Objective functions can only be minimized in GP, this is seldom a problem given that the functions to maximize are often monomials that can be inverted: a variable, a reaction rate or a flux ratio. Posynomial objectives are usually entitled for minimization, like the sum of certain variables. Nonetheless, it is also relevant in metabolic engineering to consider the maximization of posynomials, such as the sum of variables or fluxes. In such cases, condensation or recasting can be used. For en extensive introduction on GP modelling see [25].

#### A local approach: Controlled Error Method

The steady-state equation of a GMA system may be written as the single difference of two posynomials:

*P*(**x**) - *Q*(**x**) = 0

If both posynomials are condensed, every equation will be reduced to the standard form for monomial equations:

C^(P(x))C^(Q(x))=1
 MathType@MTEF@5@5@+=feaafiart1ev1aaatCvAUfKttLearuWrP9MDH5MBPbIqV92AaeXatLxBI9gBaebbnrfifHhDYfgasaacH8akY=wiFfYdH8Gipec8Eeeu0xXdbba9frFj0=OqFfea0dXdd9vqai=hGuQ8kuc9pgc9s8qqaq=dirpe0xb9q8qiLsFr0=vr0=vr0dc8meaabaqaciaacaGaaeqabaqabeGadaaakeaadaWcaaqaaiqbdoeadzaajaGaeiikaGIaemiuaaLaeiikaGccbeGae8hEaGNaeiykaKIaeiykaKcabaGafm4qamKbaKaacqGGOaakcqWGrbqucqGGOaakcqWF4baEcqGGPaqkcqGGPaqkaaGaeyypa0JaeGymaedaaa@3D00@

Because the division of a monomial by another is itself a monomial.

Since the steady state equations of the GMA have been condensed to those of an s-system, this method could be regarded as a direct generalization of classical IOM methods. One of the advantages of this approach is the possibility of keeping posynomial inequalities and objectives as they are and therefore reduce the amount of condensation (approximation) needed, but there is another interesting possibility. When a posynomial is approximated by condensation, the A-G inequality, Eq. 30, guarantees that the monomial is an underestimation of the constraint. Furthermore, the posynomial structure is not altered when divided by a monomial so the quotient between a posynomial and its condensed form is always greater than or equal to 1 and provides the exact error as a posynomial function. Therefore the problem can be constrained to allow a maximum error per condensed constraint:

∑jδj∏kXkbj,kC^(∑jδj∏kXkbj,k)≤1+ε
MathType@MTEF@5@5@+=feaafiart1ev1aaatCvAUfKttLearuWrP9MDH5MBPbIqV92AaeXatLxBI9gBaebbnrfifHhDYfgasaacH8akY=wiFfYdH8Gipec8Eeeu0xXdbba9frFj0=OqFfea0dXdd9vqai=hGuQ8kuc9pgc9s8qqaq=dirpe0xb9q8qiLsFr0=vr0=vr0dc8meaabaqaciaacaGaaeqabaqabeGadaaakeaadaWcaaqaamaaqababaacciGae8hTdq2aaSbaaSqaaiabdQgaQbqabaGcdaqeqaqaaiabdIfaynaaDaaaleaacqWGRbWAaeaacqWGIbGydaWgaaadbaGaemOAaOMaeiilaWIaem4AaSgabeaaaaaaleaacqWGRbWAaeqaniabg+GivdaaleaacqWGQbGAaeqaniabggHiLdaakeaacuWGdbWqgaqcamaabmaabaWaaabeaeaacqWF0oazdaWgaaWcbaGaemOAaOgabeaakmaarababaGaemiwaG1aa0baaSqaaiabdUgaRbqaaiabdkgaInaaBaaameaacqWGQbGAcqGGSaalcqWGRbWAaeqaaaaaaSqaaiabdUgaRbqab0Gaey4dIunaaSqaaiabdQgaQbqab0GaeyyeIuoaaOGaayjkaiaawMcaaaaacqGHKjYOcqaIXaqmcqGHRaWkcqWF1oqzaaa@57AB@

So the original problem is solved as a series of GPs in which the GMA equations are successively condensed using the previous solution as the reference point. To assure validity an extra set of constraints is added to ensure that every iteration will only explore the neighborhood of the feasible region in which error due to condensation remains below an arbitrary tolerance set by the user.

#### A global approach: Penalty Treatment

A similar yet distinct strategy that minimizes the use of condensation is an extension of the penalty treatment method [26], a classic algorithm for signomial programming. In this method, a signomial constraint such as

*P*(**x**) - *Q*(**x**) = 0

where P and Q are posynomials, is replaced by two posynomial equalities through the creation of an ancilliary variable t:

P(x)=tQ(x)=t
 MathType@MTEF@5@5@+=feaafiart1ev1aaatCvAUfKttLearuWrP9MDH5MBPbIqV92AaeXatLxBI9gBaebbnrfifHhDYfgasaacH8akY=wiFfYdH8Gipec8Eeeu0xXdbba9frFj0=OqFfea0dXdd9vqai=hGuQ8kuc9pgc9s8qqaq=dirpe0xb9q8qiLsFr0=vr0=vr0dc8meaabaqaciaacaGaaeqabaqabeGadaaakeaafaqabeGabaaabaGaemiuaaLaeiikaGccbeGae8hEaGNaeiykaKIaeyypa0JaemiDaqhabaGaemyuaeLaeiikaGIae8hEaGNaeiykaKIaeyypa0JaemiDaqhaaaaa@3A53@

These are not valid GP constraints, so the following relaxed version is used:

P(x)≤tQ(x)≤t
 MathType@MTEF@5@5@+=feaafiart1ev1aaatCvAUfKttLearuWrP9MDH5MBPbIqV92AaeXatLxBI9gBaebbnrfifHhDYfgasaacH8akY=wiFfYdH8Gipec8Eeeu0xXdbba9frFj0=OqFfea0dXdd9vqai=hGuQ8kuc9pgc9s8qqaq=dirpe0xb9q8qiLsFr0=vr0=vr0dc8meaabaqaciaacaGaaeqabaqabeGadaaakeaafaqabeGabaaabaGaemiuaaLaeiikaGccbeGae8hEaGNaeiykaKIaeyizImQaemiDaqhabaGaemyuaeLaeiikaGIae8hEaGNaeiykaKIaeyizImQaemiDaqhaaaaa@3BB1@

Upon dividing by *t*, the feasible area of the original problem is contained in the feasible area of the new relaxed version and aproximation by condensation is not needed. In order to force these inequalities to be tight in the final solution, the objective function is augmented with penalty terms that grow with the slackness of the constraints, namely the inverses of the condensation of the relaxed constraints. The result of this procedure is a legal GP:

     min⁡P0(x)+∑(wi+tC^[Pi(x)]+wi−tC^[Qi(x)])subject to:Pi(x)t≤1Qi(x)t≤1i=0⋯n
 MathType@MTEF@5@5@+=feaafiart1ev1aaatCvAUfKttLearuWrP9MDH5MBPbIqV92AaeXatLxBI9gBaebbnrfifHhDYfgasaacH8akY=wiFfYdH8Gipec8Eeeu0xXdbba9frFj0=OqFfea0dXdd9vqai=hGuQ8kuc9pgc9s8qqaq=dirpe0xb9q8qiLsFr0=vr0=vr0dc8meaabaqaciaacaGaaeqabaqabeGadaaakeGacaaygaauhuaabeqaemaaaaqaceGaYAGaaCzcaiaaxMaacaWLjaGagiyBa0MaeiyAaKMaeiOBa4gabaGaemiuaa1aaSbaaSqaaiabicdaWaqabaGccqGGOaakieqacqWF4baEcqGGPaqkcqGHRaWkdaaeabqaamaabmaabaGaem4DaC3aa0baaSqaaiabdMgaPbqaaiabgUcaRaaakmaalaaabaGaemiDaqhabaGafm4qamKbaKaacqGGBbWwcqWGqbaudaWgaaWcbaGaemyAaKgabeaakiabcIcaOiab=Hha4jabcMcaPiabc2faDbaacqGHRaWkcqWG3bWDdaqhaaWcbaGaemyAaKgabaGaeyOeI0caaOWaaSaaaeaacqWG0baDaeaacuWGdbWqgaqcaiabcUfaBjabdgfarnaaBaaaleaacqWGPbqAaeqaaOGaeiikaGIae8hEaGNaeiykaKIaeiyxa0faaaGaayjkaiaawMcaaaWcbeqab0GaeyyeIuoaaOqaaaqaceGa0AGaaCzcaiabbohaZjabbwha1jabbkgaIjabbQgaQjabbwgaLjabbogaJjabbsha0jabbccaGiabbsha0jabb+gaVjabcQda6aqaaaqaaaqaaaqaamaalaaabaGaemiuaa1aaSbaaSqaaiabdMgaPbqabaGccqGGOaakcqWF4baEcqGGPaqkaeaacqWG0baDaaGaeyizImQaeGymaedabaaabaaabaWaaSaaaeaacqWGrbqudaWgaaWcbaGaemyAaKgabeaakiabcIcaOiab=Hha4jabcMcaPaqaaiabdsha0baacqGHKjYOcqaIXaqmaeaacqWGPbqAcqGH9aqpcqaIWaamcqWIVlctcqWGUbGBaaaaaa@8756@

Where the condensed terms are calculated at the basal steady state. If the obtained solution falls within the feasible area of the original problem, it is taken as a solution, if it does not (any of the relaxed inequalities is below 1, the solution is used as the next reference point: condensations are calculated again, the weights of the violated constraints are increased and the new problem is solved. This procedure is repeated until a satisfactory solution is obtained. The original method used 1 as the initial value of the weights and increased them all in every iteration, some modifications are useful for our purposes:

• The initial weights are selected such that the overall penalty terms are just a fraction of the total objective in the initial point. In the case studies explored in this paper, such fraction was 10%.

• The weights are only increased if their corresponding constraint was violated in the last iteration. In such cases, the weight would be multiplied times a fixed value. For the case studies considered here, the choice in the value of such multiplier didn't have a significant impact in the performance of the method.

These variations on the original method serve to prevent the penalty terms from dominating the objective function and pushing the relaxed problem towards the boundaries of the feasible region from the very beginning.

### Case studies

In order to illustrate the combination of GP with BST, some optimization tasks were explored. The first example demonstrates the procedure with a very simple two variable GMA system. The second example is a model of the anaerobic fermentation pathway in *Saccharomyces cerevisiae*. The third example revisits an earlier case study concerned with the tryptophan operon in *E. coli*. These systems were optimized using the Matlab based solver ggplab [23] running on an ordinary laptop (1.6 GHz Pentium centrino, 512 Mb RAM). Matlab scripts were written in order to perform all the transformations required by the two methods described. For comparison, the models were also optimized using IOM [10] as well as Matlab's optimization toolbox. The function used in this toolbox, fmincon(), is based on an iterative algorithm called *Sequential Quadratic Programming*, which uses the BGFS formula to update the estimated Hessian matrix during every iteration [27,28].

#### A seemingly simple problem

**Figure 1:**
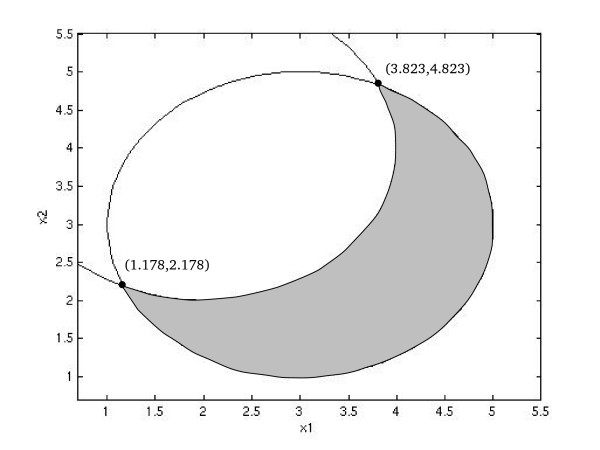
Feasible area of the first example. The lines show the nullclines of each of the two equations of the system. They intersect at two (unconnected) points, which constitute the only feasible solutions. The feasible area of the relaxed problem in the penalty treatment is marked in grey.

A very distinctive difference between the alternative methodsfor GMA optimization can be ilustrated by a problem modified from [24], which presents the simplest possible fragmented feasible region (see Fig. 1).

min⁡X1subject to:14X1+12X2−116X12−116X22−1=0114X12+114X22+1−37X1−37X2=01≤X1≤5.51≤X2≤5.5
 MathType@MTEF@5@5@+=feaafiart1ev1aaatCvAUfKttLearuWrP9MDH5MBPbIqV92AaeXatLxBI9gBaebbnrfifHhDYfgasaacH8akY=wiFfYdH8Gipec8Eeeu0xXdbba9frFj0=OqFfea0dXdd9vqai=hGuQ8kuc9pgc9s8qqaq=dirpe0xb9q8qiLsFr0=vr0=vr0dc8meaabaqaciaacaGaaeqabaqabeGadaaakeaafaqabeGbcaaaaeGabiarhiaaxMaacyGGTbqBcqGGPbqAcqGGUbGBaeaacqWGybawdaWgaaWcbaGaeGymaedabeaaaOqaceGauCGaaCzcaiabbohaZjabbwha1jabbkgaIjabbQgaQjabbwgaLjabbogaJjabbsha0jabbccaGiabbsha0jabb+gaVjabcQda6aqaaaqaaaqaamaalaaabaGaeGymaedabaGaeGinaqdaaiabdIfaynaaBaaaleaacqaIXaqmaeqaaOGaey4kaSYaaSaaaeaacqaIXaqmaeaacqaIYaGmaaGaemiwaG1aaSbaaSqaaiabikdaYaqabaGccqGHsisldaWcaaqaaiabigdaXaqaaiabigdaXiabiAda2aaacqWGybawdaqhaaWcbaGaeGymaedabaGaeGOmaidaaOGaeyOeI0YaaSaaaeaacqaIXaqmaeaacqaIXaqmcqaI2aGnaaGaemiwaG1aa0baaSqaaiabikdaYaqaaiabikdaYaaakiabgkHiTiabigdaXiabg2da9iabicdaWaqaaaqaamaalaaabaGaeGymaedabaGaeGymaeJaeGinaqdaaiabdIfaynaaDaaaleaacqaIXaqmaeaacqaIYaGmaaGccqGHRaWkdaWcaaqaaiabigdaXaqaaiabigdaXiabisda0aaacqWGybawdaqhaaWcbaGaeGOmaidabaGaeGOmaidaaOGaey4kaSIaeGymaeJaeyOeI0YaaSaaaeaacqaIZaWmaeaacqaI3aWnaaGaemiwaG1aaSbaaSqaaiabigdaXaqabaGccqGHsisldaWcaaqaaiabiodaZaqaaiabiEda3aaacqWGybawdaWgaaWcbaGaeGOmaidabeaakiabg2da9iabicdaWaqaaaqaaiabigdaXiabgsMiJkabdIfaynaaBaaaleaacqaIXaqmaeqaaOGaeyizImQaeGynauJaeiOla4IaeGynaudabaaabaGaeGymaeJaeyizImQaemiwaG1aaSbaaSqaaiabikdaYaqabaGccqGHKjYOcqaI1aqncqGGUaGlcqaI1aqnaaaaaa@8DA4@

The feasible region of this problem consists of two points (1.178,2.178) and (3.823,4.823), of which clearly the first solution is superior, because *X*_1 _is to be minimized. As these points are not connected, local methods are not able to find one solution using the other as a starting point. The problem was solved using IOM, controlled error and penalty treatment methods. The initial point was set to be (3.823,4.823), which is disconnected from the true optimal solution. While both IOM and the Controlled-Error method reported the initial point as the solution, the penalty treatment algorithm found the global optimum at (1.178,2.178).

In this case, most methods failed to find the optimal solution because the approximated s-system had the operating point as the only feasible solution while the relaxed problem for the penalty treatment algorithm had a feasible area (shadowed in Fig. 1) that included and connected both feasible solutions.

#### Anaerobic fermentation in S. cerevisiae

**Figure 2:**
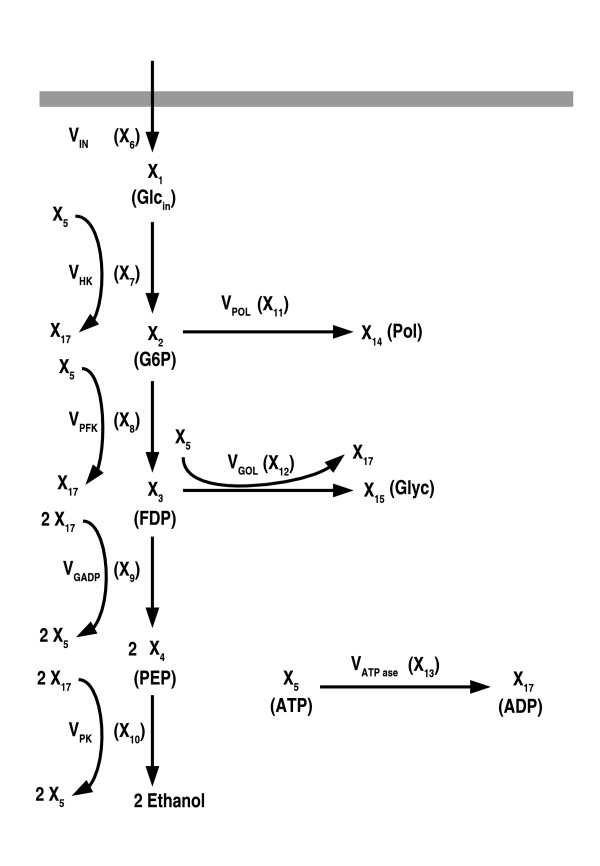
Anaerobic fermentation in *S. cerevisiae*.

This GMA model [29] (see also appendix) is derived from a previous version [30] formulated with traditional Michaelis Mentem kinetics to explain experimental data, and has been used to illustrate other optimization methods [10,17,19]. It has the following structure (see Fig. 2):

X˙1=vin−vHKX˙2=vHK−vPFK=vPOLX˙3=vPFK−vGAPD−12vGOLX˙4=2⋅vGAPD−vPKX˙5=2⋅vGAPD+vPK−vHK−vPFK−vPOL−vATP
 MathType@MTEF@5@5@+=feaafiart1ev1aaatCvAUfKttLearuWrP9MDH5MBPbIqV92AaeXatLxBI9gBaebbnrfifHhDYfgasaacH8akY=wiFfYdH8Gipec8Eeeu0xXdbba9frFj0=OqFfea0dXdd9vqai=hGuQ8kuc9pgc9s8qqaq=dirpe0xb9q8qiLsFr0=vr0=vr0dc8meaabaqaciaacaGaaeqabaqabeGadaaakeaafaqadeqbbaaaaeaacuWGybawgaGaamaaBaaaleaacqaIXaqmaeqaaOGaeyypa0JaemODay3aaSbaaSqaaiabdMgaPjabd6gaUbqabaGccqGHsislcqWG2bGDdaWgaaWcbaGaemisaGKaem4saSeabeaaaOqaaiqbdIfayzaacaWaaSbaaSqaaiabikdaYaqabaGccqGH9aqpcqWG2bGDdaWgaaWcbaGaemisaGKaem4saSeabeaakiabgkHiTiabdAha2naaBaaaleaacqWGqbaucqWGgbGrcqWGlbWsaeqaaOGaeyypa0JaemODay3aaSbaaSqaaiabdcfaqjabd+eapjabdYeambqabaaakeaacuWGybawgaGaamaaBaaaleaacqaIZaWmaeqaaOGaeyypa0JaemODay3aaSbaaSqaaiabdcfaqjabdAeagjabdUealbqabaGccqGHsislcqWG2bGDdaWgaaWcbaGaem4raCKaemyqaeKaemiuaaLaemiraqeabeaakiabgkHiTmaalaaabaGaeGymaedabaGaeGOmaidaaiabdAha2naaBaaaleaacqWGhbWrcqWGpbWtcqWGmbataeqaaaGcbaGafmiwaGLbaiaadaWgaaWcbaGaeGinaqdabeaakiabg2da9iabikdaYiabgwSixlabdAha2naaBaaaleaacqWGhbWrcqWGbbqqcqWGqbaucqWGebaraeqaaOGaeyOeI0IaemODay3aaSbaaSqaaiabdcfaqjabdUealbqabaaakeaacuWGybawgaGaamaaBaaaleaacqaI1aqnaeqaaOGaeyypa0JaeGOmaiJaeyyXICTaemODay3aaSbaaSqaaiabdEeahjabdgeabjabdcfaqjabdseaebqabaGccqGHRaWkcqWG2bGDdaWgaaWcbaGaemiuaaLaem4saSeabeaakiabgkHiTiabdAha2naaBaaaleaacqWGibascqWGlbWsaeqaaOGaeyOeI0IaemODay3aaSbaaSqaaiabdcfaqjabdAeagjabdUealbqabaGccqGHsislcqWG2bGDdaWgaaWcbaGaemiuaaLaem4ta8KaemitaWeabeaakiabgkHiTiabdAha2naaBaaaleaacqWGbbqqcqWGubavcqWGqbauaeqaaaaaaaa@9E11@

The model was already formulated [29] as a GMA system, so that all its fluxes are monomials:

vin=0.8122X2−0.2344X6vHK=2.8632X10.7464X50.0243X7vPFK=0.5232X20.7318X5−0.3941X8vGAPD=0.011X30.6159X40.1308X9X14−0.6088vPK=0.0945X30.05X40.533X5−0.0822X10vPOL=0.0009X28.6107X11vGOL=0.0945X30.05X40.533X5−0.0822X12vATP=X5X13
 MathType@MTEF@5@5@+=feaafiart1ev1aaatCvAUfKttLearuWrP9MDH5MBPbIqV92AaeXatLxBI9gBaebbnrfifHhDYfgasaacH8akY=wiFfYdH8Gipec8Eeeu0xXdbba9frFj0=OqFfea0dXdd9vqai=hGuQ8kuc9pgc9s8qqaq=dirpe0xb9q8qiLsFr0=vr0=vr0dc8meaabaqaciaacaGaaeqabaqabeGadaaakeaafaqadeacbaaaaaqaaiabdAha2naaBaaaleaacqWGPbqAcqWGUbGBaeqaaOGaeyypa0JaeGimaaJaeiOla4IaeGioaGJaeGymaeJaeGOmaiJaeGOmaiJaemiwaG1aa0baaSqaaiabikdaYaqaaiabgkHiTiabicdaWiabc6caUiabikdaYiabiodaZiabisda0iabisda0aaakiabdIfaynaaBaaaleaacqaI2aGnaeqaaaGcbaGaemODay3aaSbaaSqaaiabdIeaijabdUealbqabaGccqGH9aqpcqaIYaGmcqGGUaGlcqaI4aaocqaI2aGncqaIZaWmcqaIYaGmcqWGybawdaqhaaWcbaGaeGymaedabaGaeGimaaJaeiOla4IaeG4naCJaeGinaqJaeGOnayJaeGinaqdaaOGaemiwaG1aa0baaSqaaiabiwda1aqaaiabicdaWiabc6caUiabicdaWiabikdaYiabisda0iabiodaZaaakiabdIfaynaaBaaaleaacqaI3aWnaeqaaaGcbaGaemODay3aaSbaaSqaaiabdcfaqjabdAeagjabdUealbqabaGccqGH9aqpcqaIWaamcqGGUaGlcqaI1aqncqaIYaGmcqaIZaWmcqaIYaGmcqWGybawdaqhaaWcbaGaeGOmaidabaGaeGimaaJaeiOla4IaeG4naCJaeG4mamJaeGymaeJaeGioaGdaaOGaemiwaG1aa0baaSqaaiabiwda1aqaaiabgkHiTiabicdaWiabc6caUiabiodaZiabiMda5iabisda0iabigdaXaaakiabdIfaynaaBaaaleaacqaI4aaoaeqaaaGcbaGaemODay3aaSbaaSqaaiabdEeahjabdgeabjabdcfaqjabdseaebqabaGccqGH9aqpcqaIWaamcqGGUaGlcqaIWaamcqaIXaqmcqaIXaqmcqWGybawdaqhaaWcbaGaeG4mamdabaGaeGimaaJaeiOla4IaeGOnayJaeGymaeJaeGynauJaeGyoaKdaaOGaemiwaG1aa0baaSqaaiabisda0aqaaiabicdaWiabc6caUiabigdaXiabiodaZiabicdaWiabiIda4aaakiabdIfaynaaBaaaleaacqaI5aqoaeqaaOGaemiwaG1aa0baaSqaaiabigdaXiabisda0aqaaiabgkHiTiabicdaWiabc6caUiabiAda2iabicdaWiabiIda4iabiIda4aaaaOqaaiabdAha2naaBaaaleaacqWGqbaucqWGlbWsaeqaaOGaeyypa0JaeGimaaJaeiOla4IaeGimaaJaeGyoaKJaeGinaqJaeGynauJaemiwaG1aa0baaSqaaiabiodaZaqaaiabicdaWiabc6caUiabicdaWiabiwda1aaakiabdIfaynaaDaaaleaacqaI0aanaeaacqaIWaamcqGGUaGlcqaI1aqncqaIZaWmcqaIZaWmaaGccqWGybawdaqhaaWcbaGaeGynaudabaGaeyOeI0IaeGimaaJaeiOla4IaeGimaaJaeGioaGJaeGOmaiJaeGOmaidaaOGaemiwaG1aaSbaaSqaaiabigdaXiabicdaWaqabaaakeaacqWG2bGDdaWgaaWcbaGaemiuaaLaem4ta8KaemitaWeabeaakiabg2da9iabicdaWiabc6caUiabicdaWiabicdaWiabicdaWiabiMda5iabdIfaynaaDaaaleaacqaIYaGmaeaacqaI4aaocqGGUaGlcqaI2aGncqaIXaqmcqaIWaamcqaI3aWnaaGccqWGybawdaWgaaWcbaGaeGymaeJaeGymaedabeaaaOqaaiabdAha2naaBaaaleaacqWGhbWrcqWGpbWtcqWGmbataeqaaOGaeyypa0JaeGimaaJaeiOla4IaeGimaaJaeGyoaKJaeGinaqJaeGynauJaemiwaG1aa0baaSqaaiabiodaZaqaaiabicdaWiabc6caUiabicdaWiabiwda1aaakiabdIfaynaaDaaaleaacqaI0aanaeaacqaIWaamcqGGUaGlcqaI1aqncqaIZaWmcqaIZaWmaaGccqWGybawdaqhaaWcbaGaeGynaudabaGaeyOeI0IaeGimaaJaeiOla4IaeGimaaJaeGioaGJaeGOmaiJaeGOmaidaaOGaemiwaG1aaSbaaSqaaiabigdaXiabikdaYaqabaaakeaacqWG2bGDdaWgaaWcbaGaemyqaeKaemivaqLaemiuaafabeaakiabg2da9iabdIfaynaaBaaaleaacqaI1aqnaeqaaOGaemiwaG1aaSbaaSqaaiabigdaXiabiodaZaqabaaaaaaa@1401@

**Figure 3:**
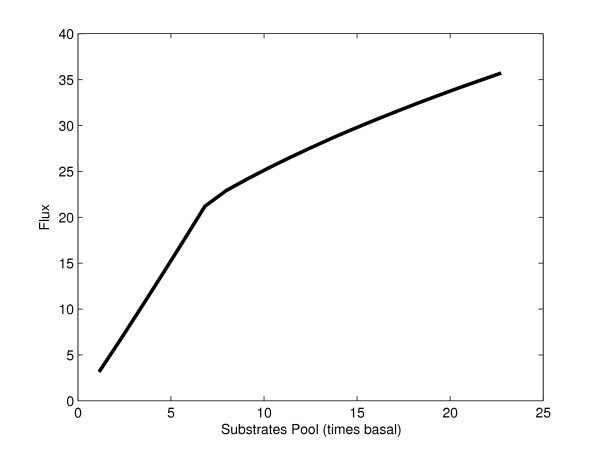
Tradeoff curve for the anaerobic fermentation pathway if the total substrate pools are kept fixed. No upper limit for total enzyme was used in this case.

The objective is (constrained) maximization of the ethanol production rate, *v*_*PK*_. Together with the upper and lower bounds of the variables, two extra constraints will be studied. The first is an upper limit to the total amount of protein. This is especially important for pathways of the central carbon metabolism as they represent a significant fraction of the total amount of cell protein and increasing the expression of its enzymes by large amounts might compromise cell viability. As a first example, we assume that the activity to protein ratio is the same for every enzyme and set an arbitrary limit of four times the amount of enzymes in the basal state. As an alternative, we explore the effect of limiting the total substrate pool. This constraint will later be subject to tradeoff analysis in order to see its influence in the optimum steady state (see Fig 3). Being posynomial functions, the constraints will be supported by GP without any transformation. The Appendix contains a complete formulation of the optimization problem.

The results are sumarized in Table 1. Both GP methods and the SQP found the same solution, although GP finished in 0.5 s while SQP was significantly slower, taking 1.5 s for the calculation. The IOM method was as fast as GP but it's solution violated one constraint.

**Table 1:** Optimization results for the GMA glycolitic model in *S. cerevisiae*. Constraint violations are shown in boldface. GP column stands for both methods

variable	basal	IOM	GP & SQP
		(times basal)	
		
*X*_1_	0.03456	2.1946	2.0000
*X*_2_	1.0110	1.5801	2.0000
*X*_3_	9.1876	1.5294	2.0000
*X*_4_	0.009532	1.1936	2.0000
*X*_5_	1.1278	**0.2803**	0.5000
*X*_6_	19.7	7.4873	7.3343
*X*_7_	68.5	3.8583	3.7794
*X*_8_	31.7	2.9176	2.8577
*X*_9_	49.9	6.4799	4.7179
*X*_10_	3440	5.7195	4.1642
*X*_11_	14.31	0.0100	0.0100
*X*_12_	203	0.0100	0.0100
*X*_13_	25.1	27.0452	14.0396
*X*_14_	0.042	1.0000	1.0000
Flux	30.2231	214.6250	198.8542

#### Tryptophan operon

**Figure 4:**
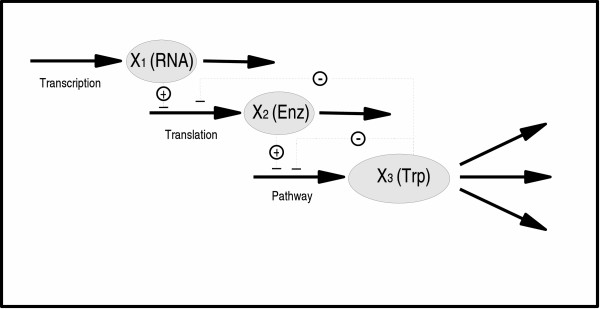
A model of the tryptophan operon. Adapted from [32].

The third example addresses the tryptophan operon in *E. coli*, as illustrated in Fig. 4. This is an appealing benchmark system, because it has already been optimized with other methods [16,31].

A model of the system was recently presented by [32] and includes transcription, translation, chemical reactions and tryptophan consumption for growth. It is thus more than a simple pathway model and demonstrates that GP and BST are applicable in more complex contexts. Finally, this model doesn't follow the structure of any standard formalism so it will be a good example on how recasting widens the applicability of the method to a higher degree of generality. The model takes the form

X˙1=v1−v2X˙2=v3−v4X˙3=v5−v6−v7−v8
 MathType@MTEF@5@5@+=feaafiart1ev1aaatCvAUfKttLearuWrP9MDH5MBPbIqV92AaeXatLxBI9gBaebbnrfifHhDYfgasaacH8akY=wiFfYdH8Gipec8Eeeu0xXdbba9frFj0=OqFfea0dXdd9vqai=hGuQ8kuc9pgc9s8qqaq=dirpe0xb9q8qiLsFr0=vr0=vr0dc8meaabaqaciaacaGaaeqabaqabeGadaaakeaafaqadeWabaaabaGafmiwaGLbaiaadaWgaaWcbaGaeGymaedabeaakiabg2da9iabdAha2naaBaaaleaacqaIXaqmaeqaaOGaeyOeI0IaemODay3aaSbaaSqaaiabikdaYaqabaaakeaacuWGybawgaGaamaaBaaaleaacqaIYaGmaeqaaOGaeyypa0JaemODay3aaSbaaSqaaiabiodaZaqabaGccqGHsislcqWG2bGDdaWgaaWcbaGaeGinaqdabeaaaOqaaiqbdIfayzaacaWaaSbaaSqaaiabiodaZaqabaGccqGH9aqpcqWG2bGDdaWgaaWcbaGaeGynaudabeaakiabgkHiTiabdAha2naaBaaaleaacqaI2aGnaeqaaOGaeyOeI0IaemODay3aaSbaaSqaaiabiEda3aqabaGccqGHsislcqWG2bGDdaWgaaWcbaGaeGioaGdabeaaaaaaaa@50B4@

Here *X*_1_, *X*_2 _and *X*_3 _are dimensionless quantities representing mRNA, enzyme levels and the tryptophan concentration, respectively. The rate equations are:

v1=X3+11+(1+X5)X3v2=(0.9+X4)X1v3=X1v4=(0.02+X4)X2v5=X2X62X62+X32v6=X3X4v7=0.0022X3X51+X3v8=(1−7.5X4)X4X3X7X3+0.005
 MathType@MTEF@5@5@+=feaafiart1ev1aaatCvAUfKttLearuWrP9MDH5MBPbIqV92AaeXatLxBI9gBaebbnrfifHhDYfgasaacH8akY=wiFfYdH8Gipec8Eeeu0xXdbba9frFj0=OqFfea0dXdd9vqai=hGuQ8kuc9pgc9s8qqaq=dirpe0xb9q8qiLsFr0=vr0=vr0dc8meaabaqaciaacaGaaeqabaqabeGadaaakeaafaqadeacbaaaaaqaaiabdAha2naaBaaaleaacqaIXaqmaeqaaOGaeyypa0ZaaSaaaeaacqWGybawdaWgaaWcbaGaeG4mamdabeaakiabgUcaRiabigdaXaqaaiabigdaXiabgUcaRiabcIcaOiabigdaXiabgUcaRiabdIfaynaaBaaaleaacqaI1aqnaeqaaOGaeiykaKIaemiwaG1aaSbaaSqaaiabiodaZaqabaaaaaGcbaGaemODay3aaSbaaSqaaiabikdaYaqabaGccqGH9aqpcqGGOaakcqaIWaamcqGGUaGlcqaI5aqocqGHRaWkcqWGybawdaWgaaWcbaGaeGinaqdabeaakiabcMcaPiabdIfaynaaBaaaleaacqaIXaqmaeqaaaGcbaGaemODay3aaSbaaSqaaiabiodaZaqabaGccqGH9aqpcqWGybawdaWgaaWcbaGaeGymaedabeaaaOqaaiabdAha2naaBaaaleaacqaI0aanaeqaaOGaeyypa0JaeiikaGIaeGimaaJaeiOla4IaeGimaaJaeGOmaiJaey4kaSIaemiwaG1aaSbaaSqaaiabisda0aqabaGccqGGPaqkcqWGybawdaWgaaWcbaGaeGOmaidabeaaaOqaaiabdAha2naaBaaaleaacqaI1aqnaeqaaOGaeyypa0ZaaSaaaeaacqWGybawdaWgaaWcbaGaeGOmaidabeaakiabdIfaynaaDaaaleaacqaI2aGnaeaacqaIYaGmaaaakeaacqWGybawdaqhaaWcbaGaeGOnaydabaGaeGOmaidaaOGaey4kaSIaemiwaG1aa0baaSqaaiabiodaZaqaaiabikdaYaaaaaaakeaacqWG2bGDdaWgaaWcbaGaeGOnaydabeaakiabg2da9iabdIfaynaaBaaaleaacqaIZaWmaeqaaOGaemiwaG1aaSbaaSqaaiabisda0aqabaaakeaacqWG2bGDdaWgaaWcbaGaeG4naCdabeaakiabg2da9maalaaabaGaeGimaaJaeiOla4IaeGimaaJaeGimaaJaeGOmaiJaeGOmaiJaemiwaG1aaSbaaSqaaiabiodaZaqabaGccqWGybawdaWgaaWcbaGaeGynaudabeaaaOqaaiabigdaXiabgUcaRiabdIfaynaaBaaaleaacqaIZaWmaeqaaaaaaOqaaiabdAha2naaBaaaleaacqaI4aaoaeqaaOGaeyypa0ZaaSaaaeaacqGGOaakcqaIXaqmcqGHsislcqaI3aWncqGGUaGlcqaI1aqncqWGybawdaWgaaWcbaGaeGinaqdabeaakiabcMcaPiabdIfaynaaBaaaleaacqaI0aanaeqaaOGaemiwaG1aaSbaaSqaaiabiodaZaqabaGccqWGybawdaWgaaWcbaGaeG4naCdabeaaaOqaaiabdIfaynaaBaaaleaacqaIZaWmaeqaaOGaey4kaSIaeGimaaJaeiOla4IaeGimaaJaeGimaaJaeGynaudaaaaaaaa@A8C5@

The GMA format is obtained by defining the following ancillary variables:

X8=1+X5X9=X3+1X10=1+X8X3X11=0.9+X4X12=0.02+X4X13=X62+X32X14=X3+0.005X15=1−7.5X4
 MathType@MTEF@5@5@+=feaafiart1ev1aaatCvAUfKttLearuWrP9MDH5MBPbIqV92AaeXatLxBI9gBaebbnrfifHhDYfgasaacH8akY=wiFfYdH8Gipec8Eeeu0xXdbba9frFj0=OqFfea0dXdd9vqai=hGuQ8kuc9pgc9s8qqaq=dirpe0xb9q8qiLsFr0=vr0=vr0dc8meaabaqaciaacaGaaeqabaqabeGadaaakeaafaqadeacbaaaaaqaaiabdIfaynaaBaaaleaacqaI4aaoaeqaaOGaeyypa0JaeGymaeJaey4kaSIaemiwaG1aaSbaaSqaaiabiwda1aqabaaakeaacqWGybawdaWgaaWcbaGaeGyoaKdabeaakiabg2da9iabdIfaynaaBaaaleaacqaIZaWmaeqaaOGaey4kaSIaeGymaedabaGaemiwaG1aaSbaaSqaaiabigdaXiabicdaWaqabaGccqGH9aqpcqaIXaqmcqGHRaWkcqWGybawdaWgaaWcbaGaeGioaGdabeaakiabdIfaynaaBaaaleaacqaIZaWmaeqaaaGcbaGaemiwaG1aaSbaaSqaaiabigdaXiabigdaXaqabaGccqGH9aqpcqaIWaamcqGGUaGlcqaI5aqocqGHRaWkcqWGybawdaWgaaWcbaGaeGinaqdabeaaaOqaaiabdIfaynaaBaaaleaacqaIXaqmcqaIYaGmaeqaaOGaeyypa0JaeGimaaJaeiOla4IaeGimaaJaeGOmaiJaey4kaSIaemiwaG1aaSbaaSqaaiabisda0aqabaaakeaacqWGybawdaWgaaWcbaGaeGymaeJaeG4mamdabeaakiabg2da9iabdIfaynaaDaaaleaacqaI2aGnaeaacqaIYaGmaaGccqGHRaWkcqWGybawdaqhaaWcbaGaeG4mamdabaGaeGOmaidaaaGcbaGaemiwaG1aaSbaaSqaaiabigdaXiabisda0aqabaGccqGH9aqpcqWGybawdaWgaaWcbaGaeG4mamdabeaakiabgUcaRiabicdaWiabc6caUiabicdaWiabicdaWiabiwda1aqaaiabdIfaynaaBaaaleaacqaIXaqmcqaI1aqnaeqaaOGaeyypa0JaeGymaeJaeyOeI0IaeG4naCJaeiOla4IaeGynauJaemiwaG1aaSbaaSqaaiabisda0aqabaaaaaaa@8070@

which turns the rates into power laws:

v1=X9X10−1v2=X11X1v3=X1v4=X12X2v5=X2X62X13−1v6=X3X4v7=0.0022X3X5X9−1v8=X15X3X7X14−1
 MathType@MTEF@5@5@+=feaafiart1ev1aaatCvAUfKttLearuWrP9MDH5MBPbIqV92AaeXatLxBI9gBaebbnrfifHhDYfgasaacH8akY=wiFfYdH8Gipec8Eeeu0xXdbba9frFj0=OqFfea0dXdd9vqai=hGuQ8kuc9pgc9s8qqaq=dirpe0xb9q8qiLsFr0=vr0=vr0dc8meaabaqaciaacaGaaeqabaqabeGadaaakeaafaqadeacbaaaaaqaaiabdAha2naaBaaaleaacqaIXaqmaeqaaOGaeyypa0JaemiwaG1aaSbaaSqaaiabiMda5aqabaGccqWGybawdaqhaaWcbaGaeGymaeJaeGimaadabaGaeyOeI0IaeGymaedaaaGcbaGaemODay3aaSbaaSqaaiabikdaYaqabaGccqGH9aqpcqWGybawdaWgaaWcbaGaeGymaeJaeGymaedabeaakiabdIfaynaaBaaaleaacqaIXaqmaeqaaaGcbaGaemODay3aaSbaaSqaaiabiodaZaqabaGccqGH9aqpcqWGybawdaWgaaWcbaGaeGymaedabeaaaOqaaiabdAha2naaBaaaleaacqaI0aanaeqaaOGaeyypa0JaemiwaG1aaSbaaSqaaiabigdaXiabikdaYaqabaGccqWGybawdaWgaaWcbaGaeGOmaidabeaaaOqaaiabdAha2naaBaaaleaacqaI1aqnaeqaaOGaeyypa0JaemiwaG1aaSbaaSqaaiabikdaYaqabaGccqWGybawdaqhaaWcbaGaeGOnaydabaGaeGOmaidaaOGaemiwaG1aa0baaSqaaiabigdaXiabiodaZaqaaiabgkHiTiabigdaXaaaaOqaaiabdAha2naaBaaaleaacqaI2aGnaeqaaOGaeyypa0JaemiwaG1aaSbaaSqaaiabiodaZaqabaGccqWGybawdaWgaaWcbaGaeGinaqdabeaaaOqaaiabdAha2naaBaaaleaacqaI3aWnaeqaaOGaeyypa0JaeGimaaJaeiOla4IaeGimaaJaeGimaaJaeGOmaiJaeGOmaiJaemiwaG1aaSbaaSqaaiabiodaZaqabaGccqWGybawdaWgaaWcbaGaeGynaudabeaakiabdIfaynaaDaaaleaacqaI5aqoaeaacqGHsislcqaIXaqmaaaakeaacqWG2bGDdaWgaaWcbaGaeGioaGdabeaakiabg2da9iabdIfaynaaBaaaleaacqaIXaqmcqaI1aqnaeqaaOGaemiwaG1aaSbaaSqaaiabiodaZaqabaGccqWGybawdaWgaaWcbaGaeG4naCdabeaakiabdIfaynaaDaaaleaacqaIXaqmcqaI0aanaeaacqGHsislcqaIXaqmaaaaaaaa@8B0D@

The objective function consists simply of *v*_8_, which may be regarded as an aggregate term for growth and tryptophan excretion.

A recurrent feature of previously found IOM solutions was the noticeable violation of a constraint retaining a minimum tryptophan concentration. This discrepancy is a feature for comparisons between methods beyond computational efficiency. The Appendix contains a complete formulation of the optimization problem.

In order to test the effectiveness of the controlled error approach, two variants were used in this model:

• Fixed tolerance. The standard method in which every iteration is limited to a maximum condensation error of 10% by constraints described in Eq. 33.

• Fixed step. No limit on the condensation error. The variation of the variables in every iteration is limited to 10% distance from the reference state.

**Figure 5:**
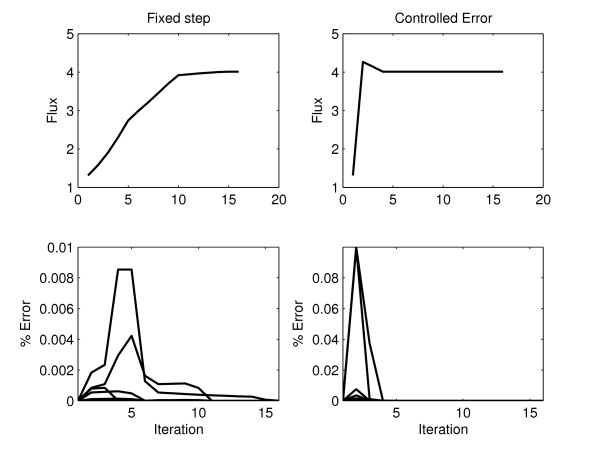
Effect of the error constraints in the optimization algorithm. Results of optimizing the model of the tryptophan operon using fixed step and fixed tolerance.

When the constraints were absent (fixed step), the variation of the variables was restricted to a fraction of the total range in every iteration, in order to prevent them from moving too far from the operating point. Fig. 5 shows the evolution of the objective function and condensation errors through iterations, both for fixed step and fixed tolerance. Though both methods find the same solution, the fixed tolerance method is much faster and keeps the error within a limit specified *a priori*. The fixed step method remains within a lower margin of error in this case due to the good quality of the condensed approximation but this margin is not under direct control and will depend on the size of the subintervals and on the model in an unforeseeable way. When the error tolerance was lowered to match the values observed for the fixed step method, both performed very similarly with a slight advantage of the fixed tolerance.

**Figure 6:**
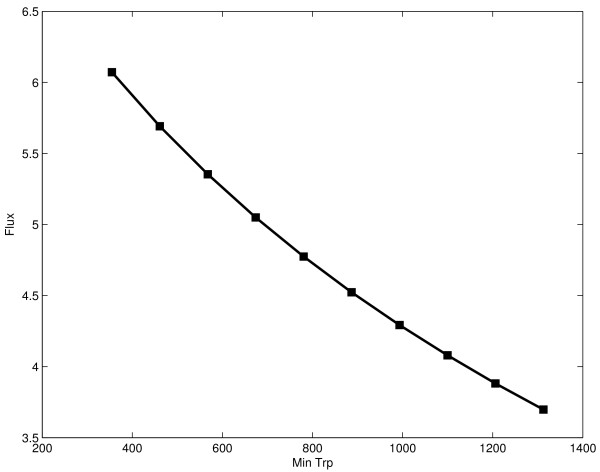
Tradeoff analysis for tryptophan model showing flux against lower bound for tryptophan.

Both the controlled error and penalty treatment methods yielded the same results while SQP returned a solution that was feasible but yielded a lower flux. As can be seen in Table 2 no constraint violations occurred with GP. When the lower bound was extended to include the levels reached by other methods, all previous results were reproduced. The tradeoff curve resulting from solving the problem for different tryptophan lower bounds is depicted as Fig 6. SQP and error controlled method took about 1 s to find the solution while the penalty tratment took 0.3 s.

**Table 2:** Comparison of results obtained for the tryptophan model with different methods. All the results that violate the lower bound for *X*_3 _were reproduced with GP by relaxing such bound. Constraint violations are shown in boldface.

			iterative	Modified		
	basal	IOM	IOM	IOM	GP	SQP
*X*_1_	0.18465	1.198 |*X*_1_|_0_	1.198 |*X*_1_|_0_	1.198 |*X*_1_|_0_	1.199 |*X*_1_|_0_	1.2 |*X*_1_|_0_
*X*_2_	7.9868	1.071 |*X*_2_|_0_	1.095 |*X*_2_|_0_	1.055 |*X*_2_|_0_	1.148 |*X*_2_|_0_	1.180 |*X*_2_|_0_
*X*_3_	1418	**0.347 **|*X*_3_|_0_	**0.465 **|*X*_3_|_0_	**0.273 **|*X*_3_|_0_	0.8 |*X*_3_|_0_	0.825 |*X*_3_|_0_
*X*_4_	0.00312	0.0058	0.0053	0.062	0.00414	0.0035
*X*_5_	5	4	4	4	4	4
*X*_6_	2283	5000	5000	5000	5000	2384
*X*_7_	430	1000	1000	1000	1000	1000
*V*_*trp*_	1.310	4.26 |*V*_*trp*_|_0_	3.884 |*V*_*trp*_|_0_	4.54 |*V*_*trp*_|_0_	3.062 |*V*_*trp*_|_0_	2.61 |*V*_*trp*_|_0_

## Conclusion

The main challenge of non-linear optimization is dealing with non-convexities. In some cases, like GP, there is an elegant transformation that convexifies the problem without adding undue complexity. But this is seldom the case and dealing with non-convexities usually implies developing *ad hoc *tricks such as subdividng the system in many subsystems, finding convex relaxations of the constraints, adding extra variables or a combination of several of these strategies.

Geometric programming provides a simple and efficient tool for the optimization of biotechnological systems that takes advantage of the structural regularity and flexibility of GMA systems. In this work we have presented two different strategies to do so, of which the penalty treatment seems to be the most promising. The methods are quite general, as this treatment of GP and recasting can be applied to any rational function, which in fact include almost all rate functions used in representations of metabolic processes.

The use of geometric programming also provides a solution for the problem of constraint violations in the two strategies considered. The possibility of keeping an arbitrarily small approximation error in every iteration prevents the buildup of discrepancies in the Controlled Error Method which results in a "safer" condensation while the Penalty treatment doesn't rely on condensation to define the feasible area. It has been shown elsewhere [21] that GP can deal with big systems, and the sparse nature of the problems in metabolic engineering improves the capabilities of the approach. It is therefore reasonable to expect both strategies considered here to scale well for big problems but it is yet to be seen which one of the two behaves better in such cases.

Geometric programming is a relatively recent and active area in operations research, which implies that further improvements and refinements for the optimization of GMA systems are to be expected. But even with existing methods, the optimization of this large class of systems, which is further expanded by the technique of recasting, has become feasible for execution of moderately sized tasks even on simple desktop computers.

## A Optimization problems

**Table 3:** A.1 Anaerobic fermentation by error controlled method

min		
Subject to:		
		Steady state
		
		Error tolerances
		

**Table 4:** A.2 Anaerobic fermentation by penalty treatment

min	
Subject to:	
Steady state	
	

**Table 5:** A.3 Tryptophan by error controlled method

min		
Subject to:		
		Steady state
		
		Ancilliary variables
		
		Error tolerances
		

**Table 6:** A.4 Tryptophan penalty approach

min		
Subject to:		
		Steady state
		
		Ancilliary variables
		

## Competing interests

The author(s) declare that they have no competing interests.
